# To reperfuse or not to reperfuse: a case report of Wellens’ syndrome with suspected COVID-19 infection

**DOI:** 10.1186/s43044-020-00094-w

**Published:** 2020-09-09

**Authors:** I Gde Rurus Suryawan, Jordan Bakhriansyah, Mia Puspitasari, Parama Gandi, Ryan Enast Intan, Firas Farisi Alkaff

**Affiliations:** 1 Department of Cardiology and Vascular Medicine, Faculty of Medicine, Universitas Airlangga – Dr. Soetomo General Academic Hospital, Jl. Mayjen Prof. Dr. Moestopo No. 6-8, Surabaya, East Java 60286 Indonesia; 2 Department of Cardiology and Vascular Medicine, Husada Utama Hospital, Surabaya, Indonesia; 3 Faculty of Medicine, Universitas Surabaya, Surabaya, Indonesia; 4 Department of Pharmacology, Faculty of Medicine Universitas Airlangga, Surabaya, Indonesia

**Keywords:** Cardiac catheterization, Case reports, COVID-19, Myocardial infarctions

## Abstract

**Background:**

Wellens’ syndrome is known to be associated with left anterior descending artery occlusion that could lead to an extensive anterior wall myocardial infarction. Thus, emergency cardiac catheterization is needed. However, during coronavirus disease 2019 (COVID-19) pandemic, it is recommended for hemodynamically stable acute coronary syndrome patients with COVID-19 infection to be treated conservatively in an isolated hospital ward.

**Case presentation:**

We report an 85-year-old patient with chief complaints of typical, squeezing chest pain in the past 4 h. The patient had a high fever, dyspnea, sore throat, and fatigue for 3 days. He had previously come into contact with COVID-19 positive relatives. The patient was hemodynamically stable and pulmonary auscultation revealed coarse rales in the entire lung. Electrocardiography (ECG) evaluation during the pain episode showed non-specific ST-T changes in lead V2-V5. After sublingual nitrate was administered, ECG evaluation during the pain-free period revealed a biphasic T wave inversion in lead V2 and V3. Laboratory workup showed elevated cardiac marker and leucopenia with neutrophilia and lymphopenia. Rapid immunochromatographic test and initial severe acute respiratory syndrome coronavirus 2 (SARS-CoV-2) reverse transcription-polymerase chain reaction (RT-PCR) evaluation from nasopharyngeal swab showed negative results. However, radiographic evaluations suggest the diagnosis of COVID-19 infection. While waiting for the second RT-PCR evaluation, the patient was diagnosed with Wellens’ syndrome with suspected COVID-19 infection. The patient was treated conservatively according to national guidelines and scheduled for elective cardiac catheterization. On the third day, the patient felt better and insisted on being discharged home. Ten days after discharged, the patient died of myocardial infarction.

**Conclusion:**

Emergency cardiac catheterization should be done for patient with Wellens’ syndrome, regardless of the COVID-19 infection status.

## Background

Wellens’ syndrome was first reported in 1982 and is known to be associated with left anterior descending (LAD) artery occlusion. If left untreated, it could lead to an extensive anterior wall myocardial infarction [1]. A later study with 18 months follow-up period showed that the mortality rate was remarkably high in patients treated conservatively compared to patients treated with cardiac catheterization (26.67% vs. 0.88%) [2]. Thus, emergency cardiac catheterization is warranted in patients presenting with this syndrome.

However, during this coronavirus disease 2019 (COVID-19) pandemic, more caution is taken into consideration for all invasive procedures, including for the cardiac catheterization procedure. In patients with COVID-19 infection, the balance of staff exposure and patient benefit should be weighed carefully. Based on the patient’s risk, conservative therapy may be sufficient for non-ST-segment elevation myocardial infarction (NSTEMI) patient with COVID-19 [3]. The Indonesian Heart Association also published a national practical clinical guideline for NSTEMI patient with COVID-19. It is recommended that patients with stable hemodynamic to be treated conservatively in isolated hospital ward [4]. In this report, we present a patient with Wellens’ syndrome with suspected COVID-19 infection based on the clinical symptoms and radiographic findings. The patient was treated conservatively according to the national guideline for NSTEMI with COVID-19 infection.

## Case presentation

An 85-year-old man came to the emergency room with the chief complaint of typical, squeezing chest pain in the past 4 h. The patient also experienced diaphoresis and nausea following chest pain. In the past 3 days, the patient had a high fever, dyspnea, sore throat, and fatigue. Past medical history of type 2 diabetes mellitus or hypertension was denied. He had a history of contact with one of his relatives who tested positive for severe acute respiratory syndrome coronavirus 2 (SARS-CoV-2) based on reverse transcription-polymerase chain reaction (RT-PCR) evaluation.

**Fig. 1:**
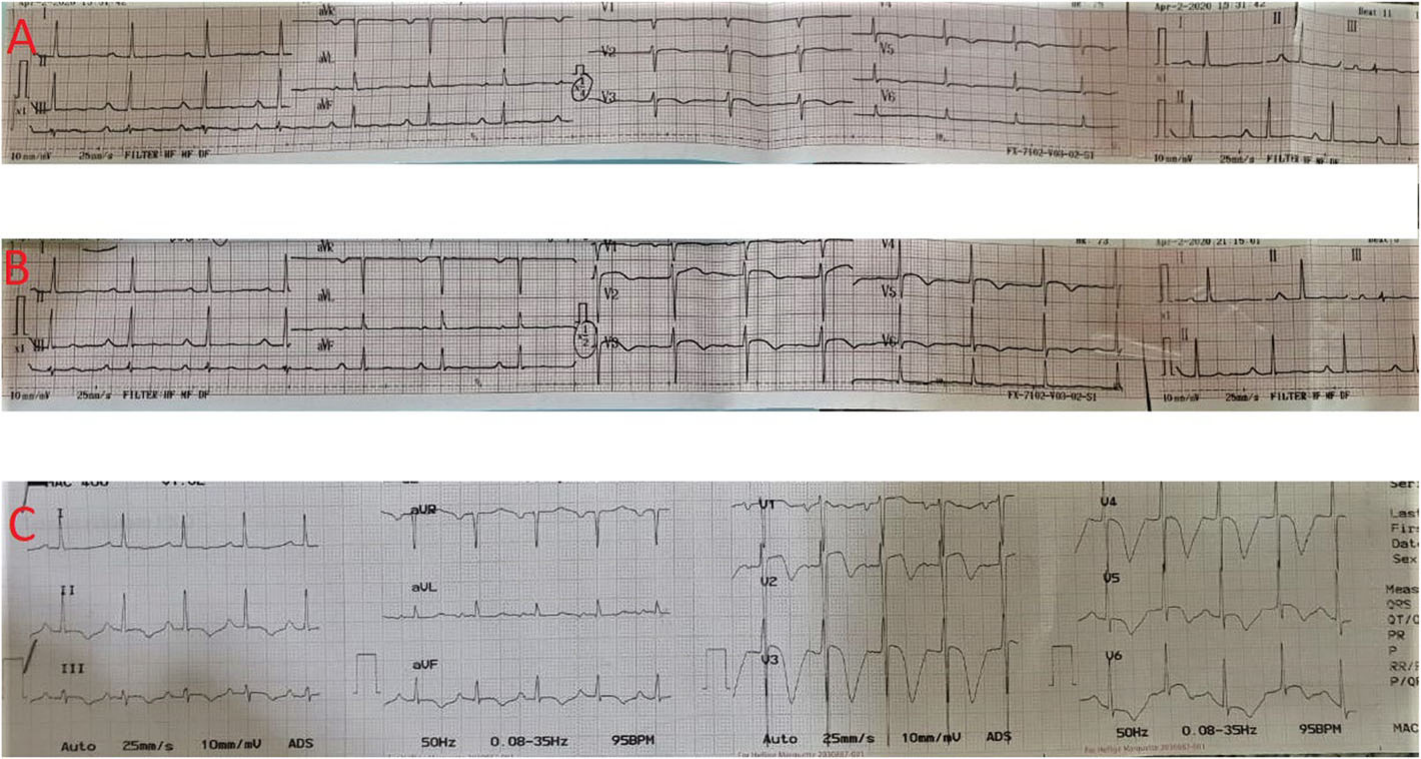
Electrocardiography evaluation (**a**) performed on admission in the emergency room during pain period showed non-specific ST-T changes in lead V2-V5, (**b**) performed on the painless period after sublingual nitrate showed biphasic T waves inversions and minimally elevated ST segment in lead V2 and V3, (**c**) performed before transferred to hospital ward during the painless period showed deeply inverted T waves in leads V2-V4

Vital signs on admission were as follows: blood pressure 130/90 mmHg, respiratory rates 26 times/min, heart rate 104 beats/min, right axillary temperature 39 °C, oxygen saturation 94% at room air, and became 99% with the simple mask with 6 L/min oxygen. Pulmonary auscultation revealed coarse rales in the entire lung. Other physical examinations were within normal limit. Twelve-lead electrocardiography (ECG) performed when the patient was in pain showed non-specific ST-T changes in lead V2-V5 (Fig. 1a). After receiving sublingual nitrate, the chest pain subsided, and the ECG evaluation showed biphasic T wave inversion and minimally elevated ST-segment in lead V2 and V3 (Fig. 1b). Before the patient was transferred to the hospital ward, the ECG evaluation in pain-free period revealed deeply inverted T waves in lead V2-V4 (Fig. 1c).

**Fig. 2:**
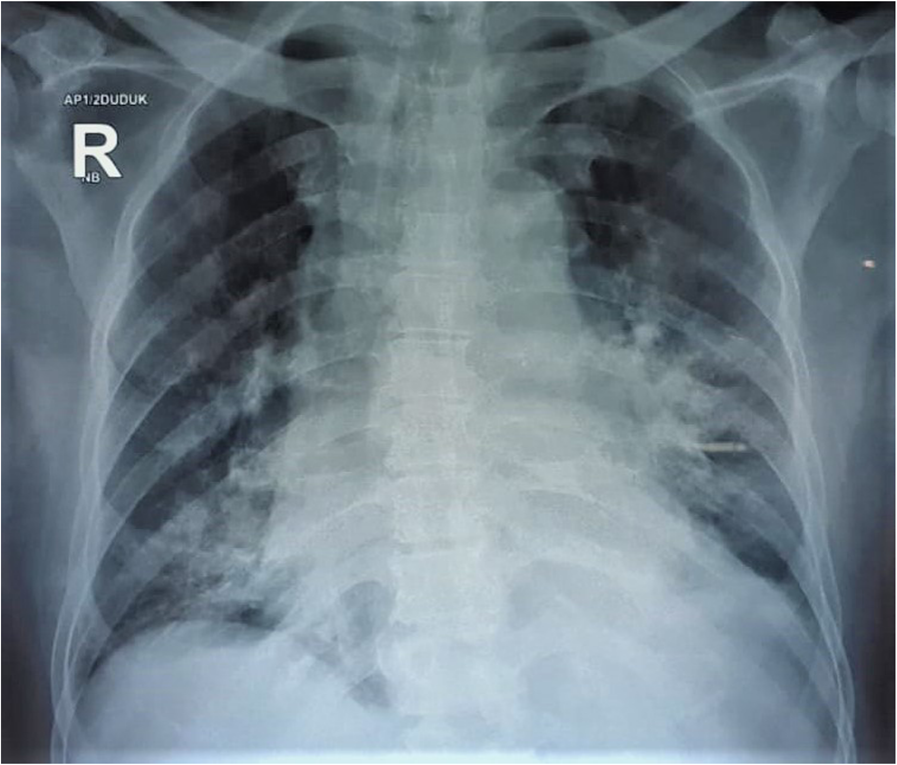
Chest X-ray showed preceding consolidation persisted with new consolidative changes in the left apical-middle-lower zone and the right lower peripheral region

**Fig. 3:**
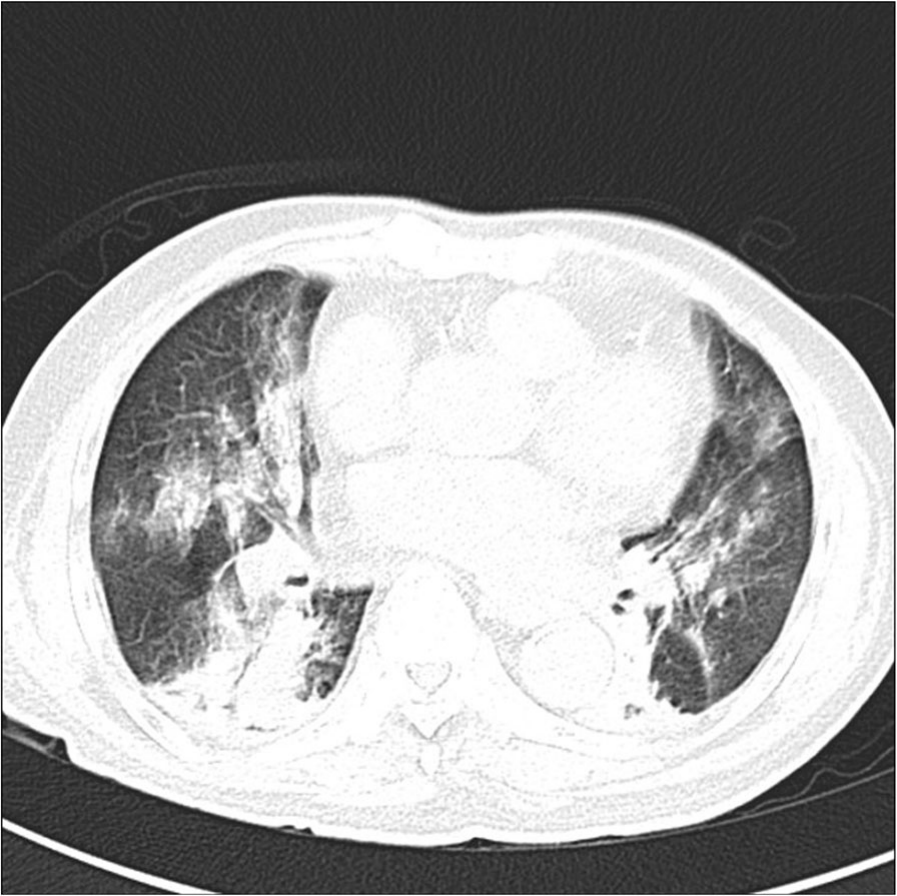
Chest computed tomography scan showed diffuse pneumonia in both lungs with multifocal ground-glass opacities and crazy paving patterns

Laboratory evaluation revealed leucopenia (3.88 × 10^3^/μl) with neutrophilia (89.4%) and lymphopenia (3.6%), thrombocytopenia (102 × 10^3^/μl), elevated aspartate transaminase (AST) (80.3 U/L), and slightly elevated alanine aminotransferase (ALT) (44.4 U/L). Creatine kinase myocardial band (CK-MB) was also increased (10.4 ng/mL). Serum creatinine and blood glass analysis were within normal limits. The Global Registry of Acute Coronary Events (GRACE) score was 159 and CRUSADE bleeding score was 37. Chest X-ray showed preceding consolidation persisted with new consolidative changes in the left apical-middle-lower zone and the right lower peripheral region (Fig. 2). Chest computed tomography scan (CT scan) revealed diffuse pneumonia in both lungs with multifocal ground-glass opacities and crazy paving patterns (Fig. 3), a common finding in patients with COVID-19 infection. However, SARS-CoV-2 rapid immunochromatographic test (Wondfo Biotech, Guangzhou, China) showed a non-reactive result. The initial RT-PCR assay (Abbott RealTime SARS-CoV-2 assay, Abbott Molecular Inc., Illinois, USA) from nasopharyngeal swab was then performed, but it also showed a negative result.

Since the diagnosis of COVID-19 infection could not be ruled out until the RT-PCR assay was repeated, the patient was diagnosed with Wellens’ syndrome with suspected COVID-19 infection. Because the patient was categorized as high-risk NSTEMI (high GRACE score but with stable hemodynamic) with high neutrophil-to-lymphocyte ratio and suspected with COVID-19 infection, the patient was treated conservatively in the intensive care unit (ICU) isolation ward while waiting for the early elective cardiac catheterization. The patient received double antiplatelet therapy (DAPT) of aspirin (80 mg once daily) and clopidogrel (75 mg once daily), fondaparinux (2.5 mg once daily), atorvastatin (80 mg once daily), bisoprolol (2.5 mg once daily), isosorbide dinitrate pump (1 mg per hour), paracetamol (500 mg thrice daily), and methisoprinol (500 mg thrice daily).

**Fig. 4:**
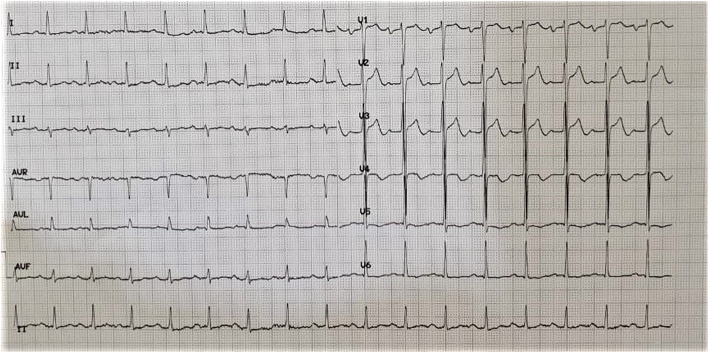
Electrocardiography re-evaluation on the third day showed biphasic T waves in lead V2 and V3

On the third day, the patient’s oxygen saturation was 98% without oxygen supplementation, and ECG evaluation reverts to biphasic T wave in lead V2 and V3 (Fig. 4). CK-MB level was still above the normal limit (19 ng/mL). The patient insisted on being discharged and refused to be referred for early elective cardiac catheterization because he already felt better. The patient and his family signed the consent form to be discharged home despite the high chance of myocardial infarction in the near future. The patient was also aware that the diagnosis of COVID-19 infection could not be ruled out yet because second RT-PCR from nasopharyngeal swab had not been performed yet, thus he and his family had to self-quarantine at home for 14 days.

On the fourth day, the patient was discharged and received aspirin (80 mg once daily) and clopidogrel (75 mg once daily) for his take-home medicine. Two weeks later, in a follow-up session via telephone, one of the family members informed that the patient already died 10 days after being discharged from our hospital due to cardiac arrest secondary to new-onset ST-elevation myocardial infarction. Due to the limited facilities in the other hospital, the patient did not undergo coronary angiography.

## Discussion

Our patient has fulfilled the suspect case criteria for COVID-19 by WHO guideline as followed: A patient with acute respiratory illness (fever and at least one sign/symptom of respiratory disease, e.g., cough, shortness of breath), AND a history of travel to or residence in a location reporting community transmission of COVID-19 disease or having been in contact with a confirmed or probable COVID-19 case during the 14 days prior to symptom onset or patients with severe acute respiratory illness (fever and at least one sign/symptom of respiratory disease, e.g., cough, shortness of breath; AND requiring hospitalization) AND in the absence of an alternative diagnosis that thoroughly explains the clinical presentation [5]. This was also supported by the laboratory abnormalities found in our patient, which were lymphopenia, leucopenia, thrombocytopenia, elevated creatinine, AST and ALT, and hypoxemia from blood gas analysis, and also pneumonia by chest X-ray and CT scan findings (bilateral, peripheral, patchy opacities on chest X-ray and bilateral ground-glass opacities, crazy paving, and multifocal consolidation from chest CT scan) that suggest high probable for COVID-19 infection. It is suggested that findings from chest CT scans usually peak around 9–13 days [6, 7].

WHO criteria for confirmed COVID-19 was based on the detection of unique sequences of virus SARS-CoV-2 RNA by nucleic acid amplification tests such as real-time RT-PCR and needed at least two positive results [8]. For initial diagnostic testing, Centers for Disease Control and Prevention (CDC) recommends collecting and testing an upper respiratory specimen with a nasopharyngeal swab as the preferred specimen choice [9]. However, multiple negative tests are required to exclude a diagnosis of COVID-19.

CDC also stated that negative SARS-CoV-2 results from RT-PCR do not preclude COVID-19 infection and should not be used as the sole basis for patient treatment decisions, especially when it is not supported with the clinical observations, patient history, and epidemiological information [9]. It is because RT-PCR has the sensitivity as low as 6-70% for initial diagnosis despite its high specificity [10]. In our case, although the initial SARS-CoV-2 RT-PCR showed a negative result, the chest CT scan showed a typical manifestation of COVID-19. This might be explained by the findings from previous study that the sensitivity of the initial chest CT scan is greater than the initial RT-PCR assay (98% vs 71%, *p* < 0.001) [11].

To establish the diagnosis of Wellens’ syndrome, it is suggested that several criteria be fulfilled, which includes (1) deep symmetrically inverted T waves or biphasic T waves in lead V2 and V3, (2) isoelectric or minimally elevated (< 1 mm) ST-segment, (3) absence of precordial Q waves, (4) history of angina, (5) pattern present during pain-free period, and (6) normal or mildly elevated creatine phosphokinase (less than two times normal upper limit) [12]. In our case, the patient fulfilled all criteria for Wellens’ syndrome except the cardiac marker. However, since the cardiac marker is known to be frequently abnormal in patients with COVID-19 [13], we argued that the cardiac marker criterion could be exempted in this situation.

There were some reports regarding the association between COVID-19 infection and cardiovascular complications including myocardial injury, myocarditis, deep vein thrombosis (DVT), and pulmonary embolism (PE) [14]. Our case might be correlated to COVID-19 infection-induced myocardial injury, infarction, or inflammation due to systemic inflammation response, marked by an elevated CK-MB level. However, it is unlikely that our patient had DVT because there were no supporting clinical findings such as warmth or pain in the extremity or asymmetrical swelling [15]. PE could also be ruled out because there was also no filling defect in the pulmonary artery in the chest CT scan evaluation [16].

It could be argued that this type of case is usually diagnosed as high-risk anterior NSTEMI. However, we would like to stress out the use of Wellens’ syndrome nomenclature to underline the high probability of total or near-total LAD occlusion that is not commonly found in high-risk NSTEMI patients. Patients with Wellens’ syndrome will develop extensive anterior wall infarction if aggressive intervention is not undertaken, despite the relief of symptoms with medical management. Half of the patients will develop the infarction within 1 week after the admission [1]. Thus, in normal situation, our patient should have undergone emergency cardiac catheterization. Other than that, our patient also had a GRACE score of 159. European Society of Cardiology (ESC) and American College of Cardiology/American Heart Association recommend an invasive strategy should be performed in less than 24 h in patient with high-risk NSTEMI (GRACE score more than 140) [17, 18].

However, in patients with suspected COVID-19 infection, the algorithm management is different. National guideline published by the Indonesian Heart Association recommends conservative treatment in the isolated hospital ward if the patients have stable hemodynamic to reduce transmission risk of COVID-19, especially when a special standardized facility is not available [4]. This recommendation was in line with the Chinese Society of Cardiology guideline that recommends patients with high-risk NSTEMI to be hospitalized and treated conservatively in designated hospital [19]. American College of Cardiology suggests that in patient with stable NSTEMI, conservative therapy may be sufficient on the basis of patient risk [3]. In contrary, guideline published by ESC recommends patients with high-risk NSTEMI to still be treated with an early invasive strategy in less than 24 h after admission in COVID-19 designated hospital [20]. According to the Egyptian Society of Cardiology guidelines, patients with high-risk NSTEMI should undergo early catheterization in less than 24 h. However, it is only possible if the hospital is not overwhelmed and all the precautions to prevent the dissemination of infection and protect the medical staff are adopted. Nevertheless, if the prevalence of COVID-19 increases and causes overburden of the health system resources, patients with high-risk NSTEMI should be hospitalized and treated conservatively in isolation wards or ICU in non-designated hospital [21].

According to the national guideline, DAPT (clopidogrel or ticagrelor and aspirin) and high-dose statin should be given for conservative treatment during hospitalization [4]. We opted to treat our patient with clopidogrel because of the moderate bleeding risk (CRUSADE score 37). Recent meta-analysis study showed that ticagrelor was associated with a higher risk of major bleeding compared to clopidogrel in East Asian patients with acute coronary syndrome [22]. In addition to those recommended treatment, the patient also received fondaparinux. After the hospitalization, the patient had been given DAPT for take-home medicine. It is recommended that DAPT should be given for 1 year after discharged home [17].

The limitations of this report were the absence of coronary angiography and echocardiography evaluation. Therefore, the diagnosis of the patients could not be confirmed and the differential diagnosis such as PE and myocarditis could not be totally excluded. The coronary angiography was not performed because it was not recommended by the Indonesian Heart Association. Echocardiography evaluation was not performed because there was no published guideline in performing echocardiography to patients with suspected COVID-19 infection and there was also a shortage of standardized personal protective equipment when this case report occurred.

## Conclusion

In the case of acute coronary syndrome in the COVID-19 pandemic situation where the risk of infectious spread is very high, risk stratification is essential to determine the treatment strategy. Following the national guideline in this situation, high-risk NSTEMI with conservative management is preferred for treatment in the acute phase, with favorable outcomes in the acute phase. However, in the case of Wellens’ syndrome, where significant LAD occlusion is suspected, urgent early cardiac catheterization should be done, regardless of the COVID-19 infection status. Recognition of the ECG pattern of Wellens’ syndrome is also crucial because Wellens’ syndrome has a poor prognosis despite the lack of symptoms in early stable conditions.

## Data Availability

Available upon request.

## Abbreviations

ALT: Alanine Aminotransferase
AST: Aspartate Transaminase
CDC: Centers for Disease Control and Prevention
CK-MB: Creatine kinase myocardial band
COVID-19: Coronavirus disease 2019
CT scan: Computed tomography scan
DAPT: Double anti platelet therapy
ECG: Electrocardiography
ESC: European Society of Cardiology
GRACE: Global Registry of Acute Coronary Events
ICU: Intensive care unit
LAD: Left anterior descending
NSTEMI: Non-ST-segment elevation myocardial infarction
RT-PCR: Reverse transcription polymerase chain reaction
SARS-CoV-2: Severe acute respiratory syndrome coronavirus 2
